# Verbal autopsy: who needs it?

**DOI:** 10.1186/1478-7954-9-19

**Published:** 2011-07-27

**Authors:** Carla AbouZahr

**Affiliations:** 1 Health Metrics Network, World Health Organization, Ave Appia, 1211 Geneva 27, Switzerland

## Commentary

Verbal autopsy has long been used to generate mortality data, often with the needs of specific programs, such as child and maternal mortality, in mind [1,2]. This led to a proliferation of instruments and the resulting data were rarely comparable across research sites or over time [3]. Demands for standardization led to the 2007 publication of the World Health Organization (WHO) verbal autopsy standards, which many researchers have adopted [4,5]. Increased convergence around standards has stimulated interest in using verbal autopsy outside research settings on a routine basis. Decision-makers, program managers, donors, and development partners have identified the need for simple data collection instruments, implemented using mobile phones or other hand-held devices and linked to the provision of care [6]. These potential users of verbal autopsy methods have different perspectives from researchers, tending to prioritize instrument simplicity, feasibility, and program relevance above technical performance. Verbal autopsy offers a solution to the challenge of generating cause of death information in settings where deaths occur outside the health care system.

The "gold standard" is medical certification of cause according to the International Classification of Diseases (ICD) [7]. The WHO verbal autopsy tools are designed to generate causes of death that are "ICD compatible." WHO recommends that mortality data derived from verbal autopsy be tabulated separately from data derived using medical certification and ICD coding [7].

While verbal autopsy may not be as reliable as hospital-based certification for identifying causes of death, it is able to produce information not available from a medical certificate. Alongside questions about signs and symptoms in the deceased person, verbal autopsy can ask about risk factors and health care seeking prior to death, elucidating social, economic, behavioral, and health system issues that may have contributed to death. This contextual knowledge is invaluable to health care managers and planners. Potential users of data generated through verbal autopsy include communities, health care planners and managers, researchers, global decision-makers, and donors [8]. While there is a degree of overlap, these users have different perspectives on the uses of mortality data. These in turn have an impact on the desirable characteristics of data collection instruments. Researchers, epidemiologists, and global-level decision-makers want mortality data to inform burden of disease estimation and program evaluation. Cause of death estimates must be scientifically validated, meet high standards of accuracy, and be comparable over time and across countries. Uncertainty in cause-specific mortality fractions can be managed.

National/subnational decision-makers and health system managers want cause of death data for planning, budgeting, and resource allocation and for monitoring and reporting to donors. The ability to track trends over time is more important than cross-country comparability. Uncertainty is problematic, especially when data are needed to inform allocation of resources. Data need to be actionable and program relevant, implying an interest in information on socio-economic determinants of mortality [9]. Data collection instruments should be feasible and cost-effective to implement and adaptable to local circumstances and conditions.

Disease-specific programs want data that highlight specific areas of interest and data collection instruments that are feasible, appropriate, and program relevant. They often have a particular interest in data on socio-economic and health system factors associated with avoidable mortality.

Beyond the health sector, civil registrars-general and national statistics offices want mortality information generated through verbal autopsy to complement data from routine administrative sources. Data collection instruments should be endorsed by technical partners such as WHO and should be simple and straightforward for implementation in routine registration encounters.

## Can one size fit all?

Given the variety of users and uses, it is legitimate to ask whether a single data collection instrument can respond to all user demands [10]. However, it would be a retrograde step to revert to a situation characterized by the coexistence of multiple, divergent tools. Instead, the research community should focus on developing methods that are aligned with core standards but can be implemented through modular approaches, adapted to local circumstances and information needs. In doing so, it is important to reflect on the potential of extending the current WHO verbal autopsy standards to incorporate socio-economic, community, behavioral, and health system determinants of mortality.

For verbal autopsy methods to successfully extend their reach from research to routine application, a solid standards-based foundation will be essential, but so will a degree of flexibility and responsiveness to user requirements. New challenges will emerge, many of which will require local-level operations and implementation research to resolve. Building the evidence base of what works and where will be critical for demonstrating to potential users that the techniques can indeed generate data that are both robust and fit for purpose. This implies rigorous validation to ensure that the data generated are reliable and scientifically sound, coupled with testing of the instruments for feasibility, sustainability, and local relevance. Both sets of criteria will need to be met if the results of verbal autopsy are to be used effectively to inform policies and programming.

## Competing interests

The author declares that they have no competing interests.
